# CD20 and CD19 promote proliferation driven by the IgM-TLR9-L265P MyD88 complex

**DOI:** 10.1093/intimm/dxaf004

**Published:** 2025-01-27

**Authors:** Yohei Kobayashi, Ryota Sato, Yuri Shimizu, Ryutaro Fukui, Takuma Shibata, Hiroki Tsukamoto, Takeshi Tsubata, Kensuke Miyake

**Affiliations:** Division of Innate Immunity, The Institute of Medical Science, The University of Tokyo; Minato-ku, Tokyo 108-8639, Japan; Division of Innate Immunity, The Institute of Medical Science, The University of Tokyo; Minato-ku, Tokyo 108-8639, Japan; Division of Innate Immunity, The Institute of Medical Science, The University of Tokyo; Minato-ku, Tokyo 108-8639, Japan; Division of Innate Immunity, The Institute of Medical Science, The University of Tokyo; Minato-ku, Tokyo 108-8639, Japan; Division of Innate Immunity, The Institute of Medical Science, The University of Tokyo; Minato-ku, Tokyo 108-8639, Japan; Department of Pharmaceutical Sciences, School of Pharmacy at Fukuoka, International University of Health and Welfare, Okawa 831-8501, Japan; Department of Pathology, Nihon University School of Dentistry, Tokyo 101-8310, Japan; Division of Innate Immunity, The Institute of Medical Science, The University of Tokyo; Minato-ku, Tokyo 108-8639, Japan

**Keywords:** AKT, B cell, IgM, lymphoma

## Abstract

The cancer driver mutation L265P MyD88 is found in approximately 30% of cases in the activated B cell-like subgroup of diffuse large B cell-like lymphoma (ABC DLBCL). L265P MyD88 forms a complex with TLR9 and IgM, referred to as the My-T-BCR complex, to drive proliferation. We here show that the B cell surface molecules CD19 and CD20 enhance proliferation mediated by the My-T-BCR complex. Using the interleukin 3 (IL-3)-dependent Ba/F3 line transduced to express the IgM complex (IgM, CD79a, and CD79b) and TLR9, we observed proliferation in the presence of anti-IgM antibody and the TLR9 ligand CpG-B. TLR9 was constitutively associated with IgM and L252P MyD88. CD19 promoted proliferation with anti-IgM and CpG-B specifically in L252P MyD88-expressing Ba/F3 cells, while CD20 enhanced the proliferation in both wild-type- and L252P MyD88-expressing Ba/F3 cells. Additionally, CD20 uniquely enabled IgM-mediated proliferation in L252P MyD88-expressing Ba/F3 cells. Although CpG-B was not required for this proliferation, TLR9 expression remained indispensable. In the ABC DLBCL line TMD8, anti-IgM antibody-mediated growth was impaired by the lack of CD20 and CD19 or of TLR9. Mechanistically, CD19 promoted IgM-dependent AKT phosphorylation, whereas CD20 increased expression of cell surface IgM, thereby enhancing the formation of the IgM-TLR9 complex. These findings suggest that CD19 and CD20 differentially contribute to the proliferation driven by the My-T-BCR complex.

## Teaser

CD20 and CD19 differentially promote B-cell lymphoma proliferation

## Introduction

The B cell receptor (BCR) signaling pathway is central to antigen-dependent B cell proliferation and differentiation into plasma cells and memory B cells (1). Beyond antigen-driven responses, BCR expression is also crucial for antigen-independent B cell homeostasis (2). Ablation of the BCR in naïve resting B cells leads to rapid cell death, demonstrating that the BCR continuously provides a tonic, antigen-independent survival signal (3). Even when protected from apoptosis through enforced expression of the anti-apoptotic molecule BCL-2, BCR-deficient B cells fail to proliferate in response to TLR ligands or CD40 ligands (4). Furthermore, the BCR signal is essential for the proliferation of B cell lymphomas (5). Diffuse large B cell lymphoma (DLBCL), the most common and aggressive form of non-Hodgkin lymphoma, often exhibits multiple genomic aberrations, including mutations in the *MYD88* and *CD79B* genes (6, 7). The L265P MYD88 mutant promotes pro-survival NF-κB signaling by forming a complex with IgM and TLR9, termed the My-T-BCR complex (2). Notably, TLR7 is unable to form a complex with IgM.

DLBCL is treated with a combination therapy known as R-CHOP, which includes the chemotherapeutic agents cyclophosphamide, doxorubicin, vincristine, and prednisone, in conjunction with the anti-CD20 monoclonal antibody (mAb) rituximab (8). CD20, a member of the MS4A family characterized by multiple transmembrane domains, is expressed on B lineage cells from the pre-B to mature B cell stages (9–11). Anti-CD20 mAbs mediate lymphoma cell killing via antibody-dependent cellular cytotoxicity and phagocytosis (ADCC/ADCP), complement-dependent cytotoxicity (CDC), and programmed cell death (PCD) (12). Although *Cd20*^*−*/*−*^ mice exhibit normal B cell development, they display impaired T cell-independent antibody responses (9, 13). In agreement with these findings, a patient with a homozygous *CD20* gene mutation demonstrates poor antibody responses to the T cell-independent antigen pneumococcal polysaccharides and the TLR9 ligand CpG (13).

CD19, a member of the immunoglobulin superfamily, is broadly expressed on B lineage cells (14). In Cd19^*−*/*−*^ mice, the B-1 cell population is reduced, although the numbers of B-2 cells remain essentially unchanged (15). CD19 is required for AKT activation upon IgM-ligation (16). Despite the clinical success of anti-CD19 chimeric antigen receptor (CAR) T cell therapy in acute lymphocytic leukemia, the role of CD19 in the proliferation of lymphoma cells driven by the My-T-BCR complex has not been fully elucidated.

To investigate how CD19 and CD20 contribute to My-T-BCR-driven proliferation, we used the interleukin 3 (IL-3)-dependent Ba/F3 cell line transduced to express the BCR complex (IgM, CD79a, and CD79b). While IgM-ligation alone did not induce proliferation in these cells, co-expression of TLR9 enabled survival and proliferation in the presence of anti-IgM antibodies and the TLR9 ligand CpG-B. Using these Ba/F3 cells, and DLBCL lines, we assessed the molecular requirements underlying My-T-BCR complex-mediated proliferation. Our findings suggest that both CD19 and CD20 promote proliferation driven by the My-T-BCR complex.

## Methods

### Cell culture

The Ba/F3 cell line was cultured in Roswell Park Memorial Institute (RPMI)-1640 medium (Nacalai Tesque) with 10% fetal bovine serum (FBS), 1 × Penicillin–Streptomycin–Glutamine (Nacalai Tesque), 50 µM β-mercaptoethanol (ME) (Nacalai Tesque) and IL-3. The TMD8 cell line and HEK293FT cell line were cultured in RPMI-1640 medium with 10% FBS, 1 × Penicillin–Streptomycin–Glutamine, 50 µM β-ME. TMD8 cell line was purchased from Creative Biolabs (Shirley, NY, USA). The OCI-LY3 cell line was purchased from DSMZ (Braunschweig, Germany) and cultured in RPMI-1640 medium with 20% FBS, 1 × Penicillin–Streptomycin–Glutamine, 50 µM β-ME. The Plat-E cell line was cultured in Dulbecco’s modified Eagle’s medium (DMEM) (high glucose) medium (Nacalai Tesque) with 10% FBS, 1 × Penicillin–Streptomycin–Glutamine, 50 µM β-ME.

### Reagents

CpG-B1668 was synthesized by FASMAC. R848 was purchased from Invivogen. AKT inhibitor (MK2206) was purchased from Selleck Chemical. The sequences of guide RNA (gRNA) used were as follows: human CD19 gRNA; TGGAATGTTTCGGACCTAGG, human CD20 gRNA; AGGAGGATGTCTTCACTGGT, human TLR9 gRNA; AAAGGCTGGTGACATTGCCA, mouse TLR9 gRNA; TCAGCTGCCGCAGGTTGGAC, mouse IgM gRNA; TGTGCAAGATACGATTACTA.

### Plasmid constructs

Original pMX4, pLCV2, and pKLV2 plasmids were purchased from Addgene. The pMX4 vectors are used for over-expression. The pLCV2 and pKLV2 vectors (with puromycin-resistant gene) are used for gRNA and hCas9 expression.

Mouse IgM and Igλ cDNA were kindly provided by Prof. T. Kurosaki (Osaka University, Osaka, Japan) and Prof. T. Tsubata (Nihon University, Tokyo, Japan). TLR9, TLR7, Unc93b1, CD79a, CD79b, CD20, CD19, and MyD88 cDNAs were amplified by PCR from mouse spleen cDNA. Human IgM cDNA was purchased from Invivogen. cDNAs were cloned into retroviral pMX4 vectors. The NEBuilder HiFi DNA Assembly Cloning Kit (New England BioLabs) and Rapid DNA Ligation Kit (Roche Applied Science) were used for the cloning.

### Antibodies

Monoclonal anti-mouse Igλ (clone RML-42), CD20 (clone SA275A11), CD79a (clone F11-172), CD79b (clone HM79-12), anti-rat CD2 (clone OX-34), anti-human IgM (clone MHM-88), CD20 (clone 2H7), TLR9 (clone S16013D), and CD19 (clone SJ25C1) antibodies were purchased from BioLegend. Monoclonal antibodies (mAbs) to mouse TLR9 (clone J15A7), TLR7 (A94B10), and CD19 (clone 1D3) were purchased from BD Biosciences. The mAbs anti-mouse IgM (clone II/41) and Protein A-HRP (#101023) were purchased from Invitrogen. The monoclonal anti-phospho-Akt (clone 193H12), phospho-NF-κB p65 (clone 93H1), phospho-Btk (clone D1D2Z), IgM (clone E8M1B), IgM (clone E9U8J), MyD88 (clone D80F5), and TLR7 (clone E4J3Z) were purchased from Cell Signaling Technology (CST). Polyclonal phospho-Syk antibody was purchased from Invitrogen. The monoclonal anti-beta Actin (clone SP124) antibody was purchased from Abcam. The mouse mAb mouse TLR9 (NaR9) and TLR7 (A94B10) were established in the Miyake laboratory.

### Retroviral transduction

The pMX4 vectors were transfected into Plat-E packaging cells with FuGene6 (Roche). After 2 days of incubation, supernatants were collected as virus suspensions. Ba/F3 cells were transduced by virus suspensions mixed with DOTAP (Roche).

### Lentiviral transduction

To establish mutant cells, the pLCV2 and pKLV2 vectors were transfected into the HEK293FT cells with ViraPower Lentiviral Expression Systems (Invitrogen). After 2 days of incubation, the supernatants were obtained as viral suspensions. Ba/F3 cells were infected by the viral suspensions. For TMD8 cells, the supernatants were incubated with Lenti-X concentrator (takara) according to the manufacturer’s instructions and then infected.

### Cell proliferation assay

Cells were planted on 48 or 96-well plates at 2 × 10^4^ per well for Ba/F3 cells and 1 × 10^4^ per well for TMD8 and OCI-LY3 cells. To remove IL-3, Ba/F3 cells were washed with PBS two times. Ba/F3 cells were stimulated by AffiniPure F(ab’)_2_ Fragment Goat Anti-Mouse IgM (Jackson ImmunoResearch), R848 (Invivogen) and CpG-B1668 (FASMAC). TMD8 and OCI-LY3 cells were stimulated by anti-human IgM Antibody (Biolegend). The cells were counted by CellTiter 96R AQueous One Solution Cell Proliferation Assay (Promega) according to the manufacturer’s instructions.

### Immunoblot analysis

The cells were lysed in Sample Buffer (50 mM Tris-HCl 10% glycerol, 1% SDS, and 10% 2-ME). The lysates were heated at 95°C for 10 min. The samples were separated using SDS-PAGE and subjected to immunoblot analysis. The antibodies used for immunoblot analysis were dissolved in CanGetSignals Solution 1 (Toyobo). The secondary antibodies used were Protein A-HRP (Invitrogen) dissolved in CanGetSignals Solution 2. The intensity of the developed bands was quantified by ImageJ software (NIH, Bethesda, MD, USA).

### Immunoprecipitation

Ba/F3 cells were lysed at 5 × 10^6^ cells per ml in a lysis buffer (1% Triton, 30 mM Tris-HCl, 150 mM NaCl, 0.5 mM CaCl_2_, 0.5 mM MgCl_2_, 10% Glycerol, 1 × complete inhibitor Cocktail (Roche)) for 30 min on ice. The lysates were separated from debris by centrifugation at 15 000 rpm for 15 min at 4°C. Ba/F3 cells lysates were added to 4FF beads [anti-mouse TLR9 mAb (NaR9) or TLR7 mAb (A94B10) conjugated] and samples were rotated for more than 2 h. After rotation, Beads were washed with wash buffer (0.1% Triton, 30 mM Tris-HCl, 150 mM NaCl, 0.5 mM CaCl_2_, 0.5 mM MgCl_2_, 10% Glycerol, 0.1 × complete inhibitor Cocktail) three times. Beads were eluted by Sample Buffer (50 mM Tris-HCl 10% glycerol, 1% SDS, and 10% 2-ME) and incubated at 95°C for 10 min. The samples were separated by SDS-PAGE and subjected to immunoblot analysis.

### Cell staining and flow cytometry

Cells were stained and analyzed in fluorescence-activated cell sorter (FACS) buffer (0.1% NaN_3_, 2.5% FBS in PBS). For permeabilized staining, Cytofix/Cytoperm Fixation/Permeabilization Kit (BD Biosciences) was applied to cells before staining the cells. Cells were analyzed with Spectral Cell Analyzer ID7000 (SONY) and the data were analyzed with FlowJo software (BD Biosciences).

### Statistical analysis

All data are represented as mean ± SEM, and graphs were generated using Prism (GraphPad Software, San Diego, CA). Differences among three or more groups were evaluated by one-way analysis of variance (ANOVA) with the Tukey post hoc test or by two-way ANOVA with the Tukey post hoc tests. *P-*values of <0.05 were considered significant.

## Results

### Ba/F3 proliferation driven by TLR9 and IgM

**Figure 1.**
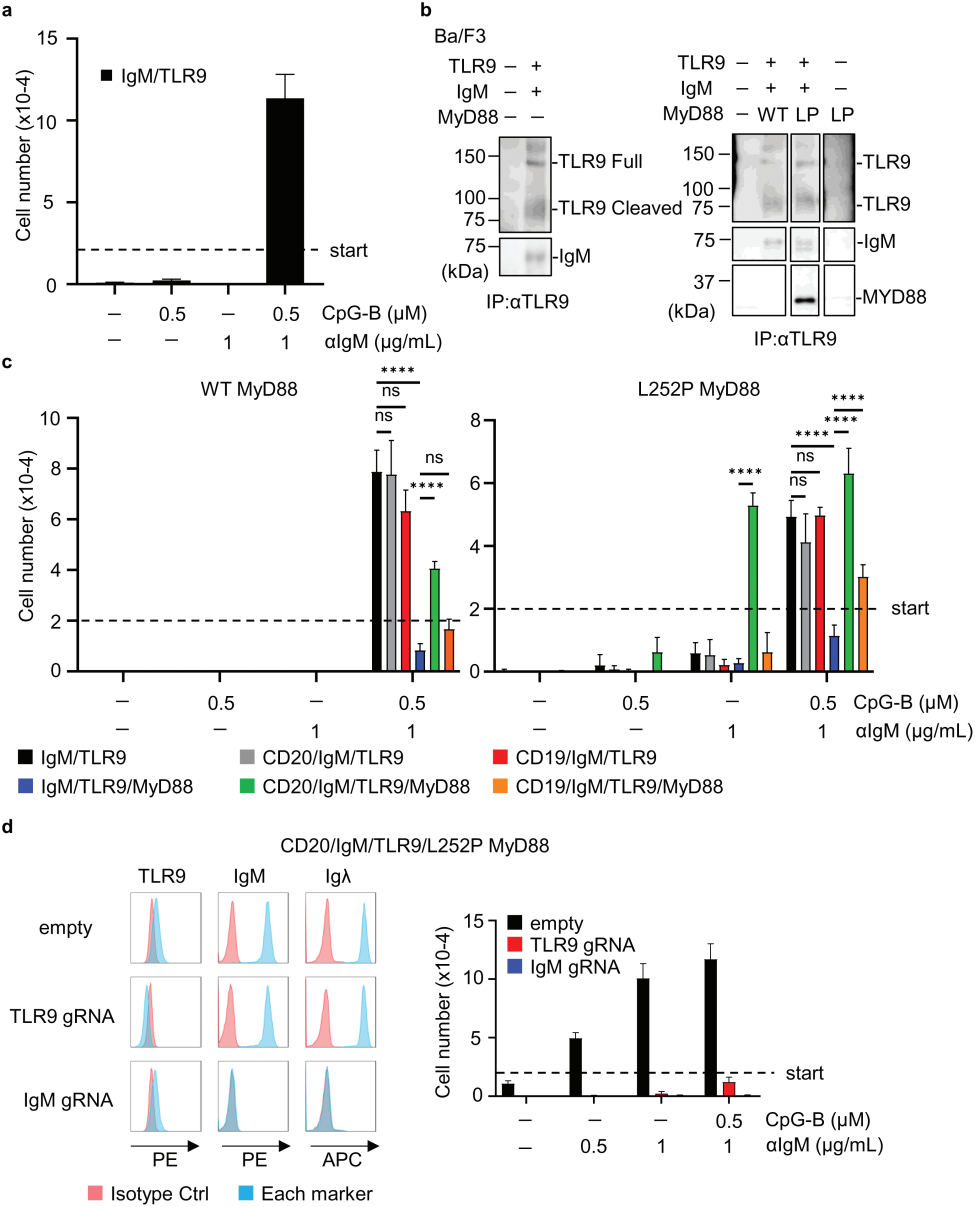
CD19 and CD20 promote L252P MyD88-dependent proliferation. (a) The numbers of IgM/TLR9-expressing Ba/F3 cells (M/T9) after the culture with indicated stimulations for 5 days. The dotted line indicates the number of cells at the beginning of the culture. Mean values ± SEM from triplicate samples are shown. (b) Immunoblot analysis of TLR9, IgM, MyD88 in the immunoprecipitates with anti-TLR9 mAb of indicated Ba/F3 cells. (c) The numbers of indicated Ba/F3 cells that were cultured with indicated stimulations for 5 days. (d) Histograms show staining of indicated Ba/F3 cells with antibodies to an indicated marker (blue) or isotype Ab (red). The right panel shows the numbers of Ba/F3 cells expressing CD20, IgM, TLR9, and L252P MyD88 with or without indicated gRNAs after the culture with indicated stimulations. These experiments were repeated twice, and representative data are shown. ****P < 0.0001.

To investigate the molecular requirements for B cell proliferation mediated by the IgM-TLR9 complex, the IL-3-dependent pro-B cell line Ba/F3 was engineered to co-express the IgM complex (μ chain, λ chain, CD79a, and CD79b) (Supplementary Figure 1a) and the TLR9 complex (TLR9 and its chaperon Unc93B1). Under these conditions, simultaneous stimulation with an anti-IgM antibody and the TLR9 ligand CpG-B induced cell proliferation in the absence of IL-3 (Fig. 1a).

### CD19 and CD20 are required for L252P MyD88-dependent proliferation

To investigate the role of MyD88 in the IgM-TLR9-dependent proliferation, we over-expressed wild-type or mutant MyD88 in Ba/F3 cells and examined IgM-TLR9-MyD88 interactions by immunoprecipitating TLR9. IgM was co-precipitated with TLR9 regardless of MyD88 over-expression (Fig. 1b). L252P MyD88 the mouse homologue of L265P MyD88, but not wild-type MyD88, was co-precipitated with TLR9 (Fig. 1b), suggesting that the L265P mutation strengthens MyD88–TLR9 interaction.

We next assessed proliferation under stimulation with anti-IgM antibody and CpG-B. Over-expression of L252P MyD88 did not induce proliferation in the absence of these stimuli (Fig. 1c, blue bar in the right panel). Furthermore, MyD88 over-expression—whether wild-type or L252P—downregulated proliferation driven by anti-IgM antibody and CpG-B (Fig. 1c, compare black and blue bars). Increased expression of NF-κB-GFP indicated that wild-type and L252P MyD88 activated downstream signaling (Supplementary Figure 1b). However, NF-κB activation by over-expressed MyD88 did not promote proliferation, suggesting that over-expressed MyD88 does not work downstream of the IgM-TLR9 complex. Constitutive NF-κB activation might inhibit ligand-dependent NF-κB activation downstream of the IgM-TLR9 complex. We hypothesized that a molecule necessary for MyD88-dependent proliferation is missing in Ba/F3 cells. Given that CD19 promotes proliferation in germinal center B cell-like DLBCL (17) and CD20 deficiency impairs TLR9 responses (13, 17), CD19 and CD20 were expressed in Ba/F3 cells to determine their role in proliferation (Supplementary Figure 1a). Neither CD19 nor CD20 altered IgM-TLR9-dependent proliferation in the absence of MyD88 over-expression (Fig. 1c, compare black vs gray/red bars). However, CD20 expression promoted proliferation with anti-IgM and CpG-B in both wild-type and L252P MyD88-expressing Ba/F3 cells (Fig. 1c, compare blue and green). CD19 enhanced this proliferation only in L252P MyD88-expressing Ba/F3 cells (Fig. 1c, compare blue and orange). Moreover, CD20, but not CD19, enabled proliferation in L252P MyD88-expressing Ba/F3 cells stimulated with anti-IgM antibody alone (Fig. 1c, green bars). This proliferation did not require CpG-B stimulation but still required TLR9, as targeting TLR9 with a specific gRNA impaired the proliferation (Fig. 1d). These findings suggest that CD20 and CD19 promote L252P mutation-dependent proliferation in Ba/F3 cells.

### CD19 promotes IgM-dependent AKT phosphorylation in Ba/F3 cells

**Figure 2.**
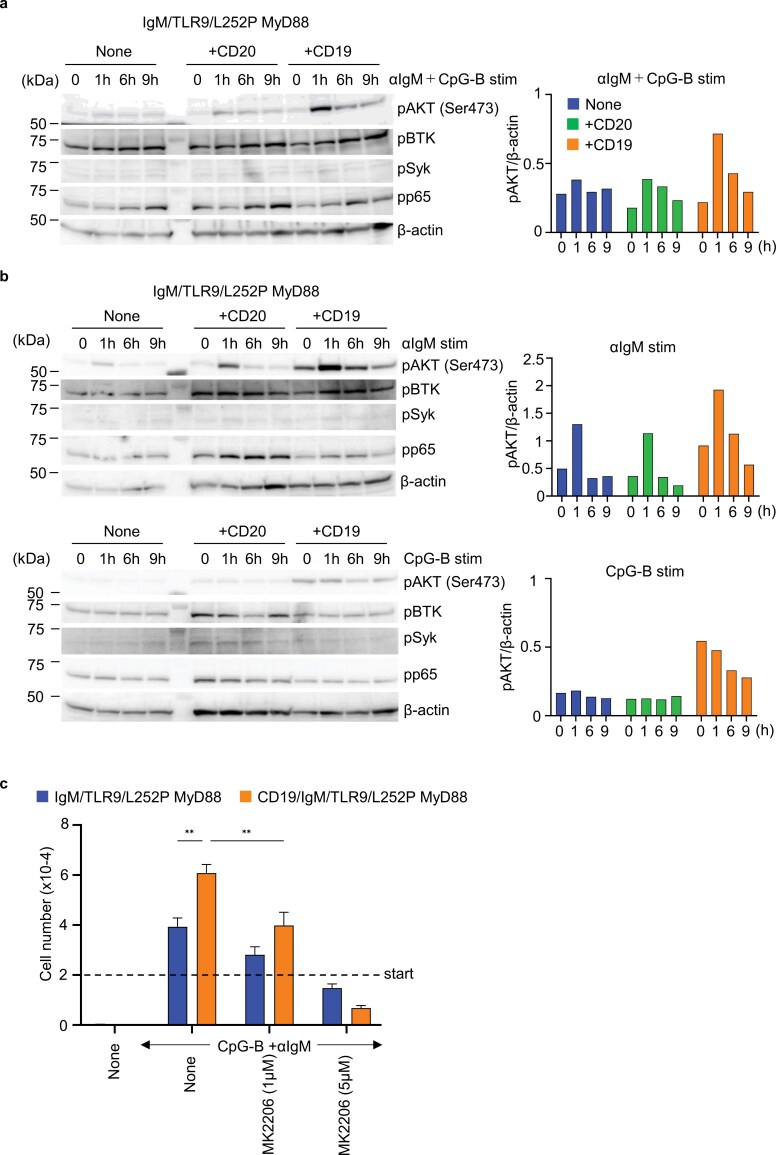
CD19 promotes AKT phosphorylation upon IgM-ligation. (a, b) Immunoblot analysis to detect phosphorylation of AKT at Ser 473, BTK, Syk, and p65 in indicated Ba/F3 cells at indicated times after stimulation with CpG-B and anti-IgM Ab (a) or anti-IgM Ab or CpG-B (b). β-actin is shown as the loading control. The intensity of the pAKT band with β-actin was quantified and plotted it on a graph. (c) The numbers of indicated Ba/F3 cells that survived the 3 days culture with indicated stimulation and indicated concentrations of AKT inhibitor MK2206. The results are represented by the mean value ± SEM from triplicates. These experiments were repeated twice, and representative data are shown. **P < 0.01.

CD19 has been reported to enhance IgM-mediated signaling (16, 18). To determine which downstream signaling pathways are strengthened by CD19 in Ba/F3 cells, we conducted biochemical analyses following stimulation with anti-IgM antibody and CpG-B. We examined the phosphorylation status of AKT, BTK, Syk, and p65. Under these conditions, Ba/F3 cells exhibited stimulation-dependent phosphorylation of AKT at ser473 (Fig. 2a). Notably, AKT phosphorylation was augmented by CD19 over-expression. Furthermore, CD19 enhanced AKT phosphorylation in response to IgM ligation alone, whereas CpG-B stimulation did not induce phosphorylation of AKT, BTK, Syk, or p65 in this Ba/F3 line (Fig. 2b). These results suggest that CD19 specifically augments IgM-dependent AKT-phosphorylation. To assess the functional relevance of AKT activation in this Ba/F3 line, we treated Ba/F3 cells with the AKT inhibitor MK2206 under anti-IgM antibody and CpG-B stimulation. Inhibition of AKT significantly reduced proliferation of CD19/IgM/TLR9/L265P MyD88-expressing Ba/F3 (Fig. 2c), suggesting that CD19-dependent AKT phosphorylation promotes the My-T-BCR complex-driven proliferation in Ba/F3 cells.

### CD20 increases cell surface IgM expression

**Figure 3.**
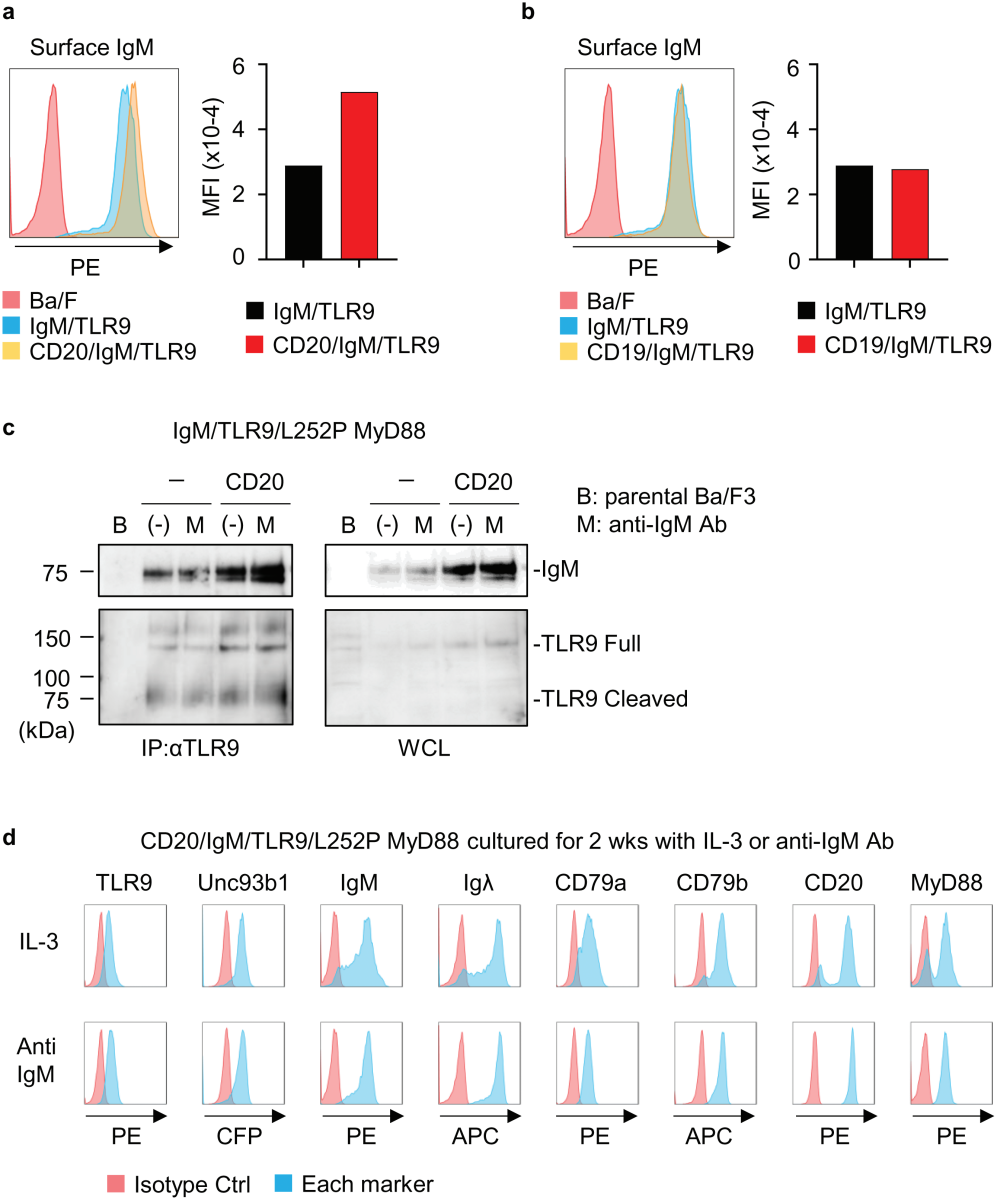
CD20 promotes cell surface expression of IgM. (a, b) Histograms (left) and mean fluorescence intensity (right) of the staining of cell surface IgM on indicated Ba/F3 cells. (c) After immunoprecipitation of TLR9, TLR9 and co-precipitated IgM were detected (left). The right panel shows immunoblotting of IgM and TLR9 in whole cell lysates from indicated cells. B, parental Ba/F3 cells. M, anti-IgM Ab. (d) Indicated Ba/F3 cells were cultured for 2 weeks in the presence of IL-3 or anti-IgM Ab. Expression of indicated molecules are shown by blue histograms. Red histograms show the staining with the isotype control Ab. These experiments were repeated twice, and representative data are shown.

We next focused on the role of CD20 in IgM/TLR9-dependent proliferation. Previous studies showed that CD20 associates with IgM on the cell surface and the association is disrupted upon IgM internalization (19, 20). In this context, we found that the amount of cell surface IgM increased by CD20 expression, but not CD19 expression (Fig. 3a, 3b). This is consistent with the previous study on *Cd20*^*−*/*−*^ mice and the B cell lymphoma line Ramos lacking CD20 (9, 18). We hypothesized that increased expression of IgM on the cell surface results in increases in the IgM-TLR9 complex. Ba/F3 cells that expressed IgM, TLR9, and L252P MyD88 with or without CD20 were left unstimulated or stimulated with anti-IgM antibody for 24 hrs. The amount of IgM in the cell lysate was apparently increased with CD20 expression (Fig. 3c). TLR9 was immunoprecipitated and coprecipitation of IgM was examined. The amount of co-precipitated IgM also increased with CD20 expression, but not with IgM-ligation. These results suggest that CD20 expression increased the amount of cell surface IgM and thereby of the IgM-TLR9 complex, probably in the endosomal compartment.

We next examined expression of IgM, TLR9, MyD88, and CD20 after *in vitro* culture with IL-3 or an anti-IgM antibody for 2wks (Fig. 3d). We found that the populations that poorly expressed the IgM complex (μ, λ, CD79a, and CD79b) and L252P MyD88 complex disappeared during the 2 wk culture with anti-IgM antibody, but not with IL-3, suggesting that only the population highly expressing IgM and L252P MyD88 survive the selection with anti-IgM antibodies. As the population poorly expressing CD20 did not survive the culture with IgM-ligation, suggesting that CD20 is as essential as the IgM complex and L252P MyD88 for survival/proliferation driven by the My-T-BCR complex.

### CD19 and CD20 promote IgM-dependent proliferation in TMD8

**Figure 4.**
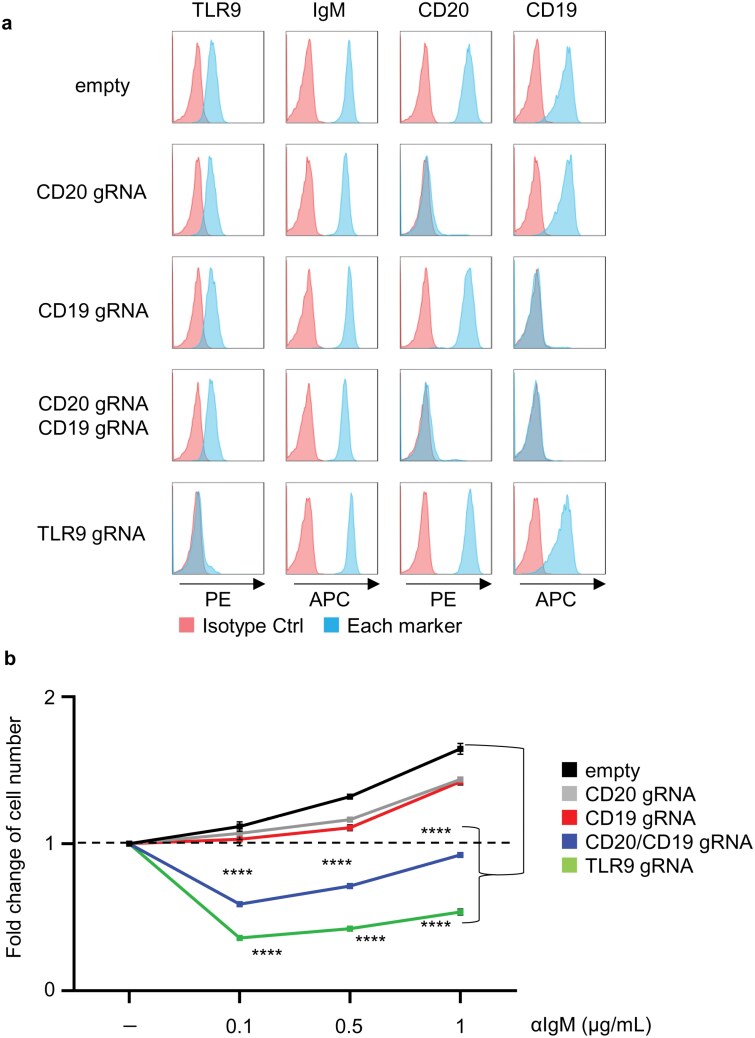
CD19 and CD20 promote IgM-dependent proliferation in the DLBCL line TMD8. (a) Blue histograms show expression of indicated markers in TMD8 cells expressing indicated gRNA. Red histograms show the staining with isotype control Ab. (b) Fold increases in the numbers of TMD8 cells expressing indicated gRNAs after 3 days culture with indicated concentrations of anti-IgM Ab. The results are represented by the averaged fold increase ± SEM from triplicates. Dotted lines indicate the fold increase as 1. These experiments were repeated twice, and representative data are shown. ****P < 0.0001.

To study the role of CD19 and CD20 in the proliferation of the activated B cell-like (ABC) subgroup of DLBCL line TMD8 (21), gRNAs targeting CD19, CD20, and TLR9 were transduced. Abolished expression of CD19, CD20, CD19 + CD20, and TLR9 was confirmed by FACS analyses (Fig. 4a). We first studied survival/proliferation at the steady state but could not obtain stable results probably because of changes in the conditions of these lines. We found that stable results were obtained by studying survival/proliferation of these cells in the presence of anti-IgM antibody (Fig. 4b). Anti-IgM antibody promoted survival/proliferation of TMD8 and the growth-promoting effect of IgM-ligation was impaired by downregulation of TLR9, suggesting that anti-IgM antibody promotes proliferation driven by the My-T-BCR complex. Downregulation of both CD19 and CD20 also impaired the growth-promoting effect of IgM-ligation (Fig. 4b). These results suggest that CD19 and CD20 promote TMD8 proliferation driven by the My-T-BCR complex.

### Impaired AKT phosphorylation upon IgM-ligation in CD19/CD20-deficient TMD8

**Figure 5.**
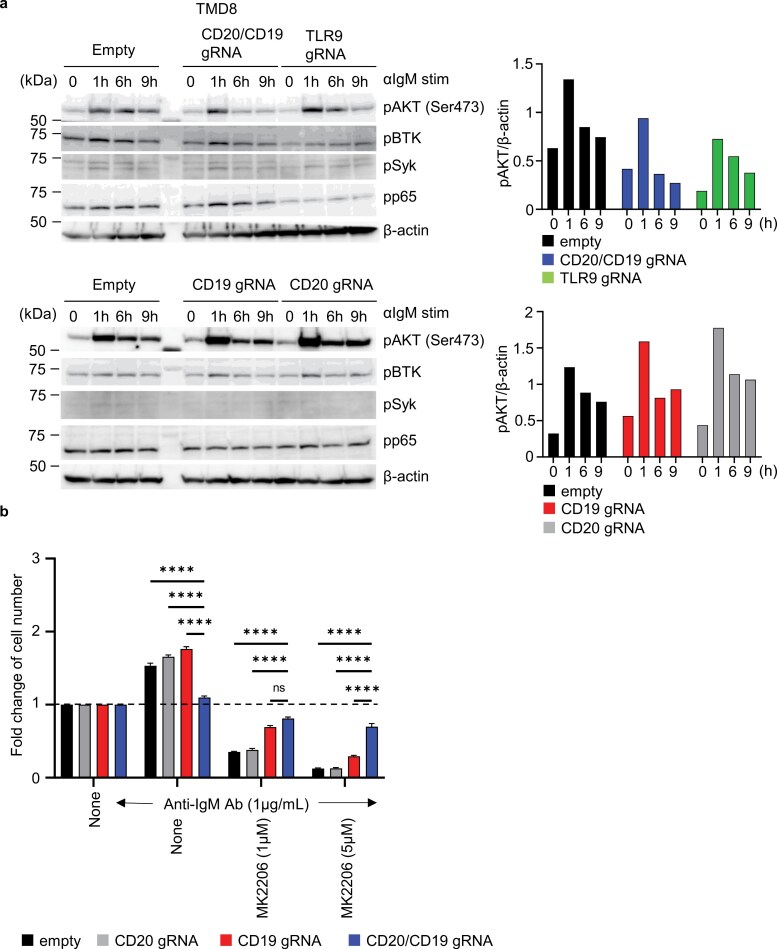
CD19 promotes AKT phosphorylation in TMD8. (a) Immunoblot analysis to detect phosphorylation of AKT at Ser 473, BTK, Syk, and p65 in indicated TMD8 cells at indicated times after stimulation with anti-IgM Ab. β-actin is shown as the loading control. The intensity of the pAKT band with β-actin was quantified and plotted it on a graph. (b) Fold increases in the numbers of TMD8 cells expressing indicated gRNAs after 3 days culture with anti-IgM Ab and indicated concentrations of the AKT inhibitor MK2206. The results are represented by the averaged fold increase ± SEM from triplicates. Dotted lines indicate the fold increase as 1. These experiments were repeated twice, and representative data are shown. ****P < 0.0001.

To delineate the roles of CD19 and CD20 in IgM-ligation-dependent AKT phosphorylation in the TMD8 line (2, 21), we first examined the CD19/CD20-deficient TMD8 line. In parental TMD8 cells, AKT phosphorylation increased 1 h after anti-IgM antibody stimulation and remained elevated at 9 h (Fig. 5a). By contrast, in CD19/CD20-deficient TMD8 cells, AKT phosphorylation increased at 1 h but declined by 9 h, indicating that the downregulation of CD19 and CD20 reduced duration of AKT activation (Fig. 5a). A similar reduction in AKT phosphorylation duration was observed in TLR9-deficient TMD8 cells, suggesting that sustained AKT phosphorylation require not only TLR9 but also CD19 and CD20 in the TMD8 line. To further assess this requirement, we examined AKT phosphorylation in CD19-deficient TMD8 and CD20-deficient TMD8 cells. In these cells, AKT phosphorylation was unaffected (Fig. 5a). We next investigated the functional importance of AKT activation in TMD8 proliferation using the AKT inhibitor MK2206. Parental and CD20-deficient TMD8 lines were more sensitive to MK2206-mediated growth inhibition than CD19/CD20-deficient TMD8 cells (Fig. 5b). CD19-deficient TMD8 cells showed intermediate sensitivity. This observation suggests that reduced AKT phosphorylation, due to the simultaneous downregulation of CD19 and CD20, might shift the survival and proliferation of TMD8 cells in response to IgM ligation toward an AKT-independent mechanism. These results suggest that both CD19 and CD20 promote TMD8 proliferation by sustaining IgM-ligation-mediated AKT phosphorylation.

### CD20 increased expression of cell surface IgM in TMD8 and OCI-Ly3

**Figure 6.**
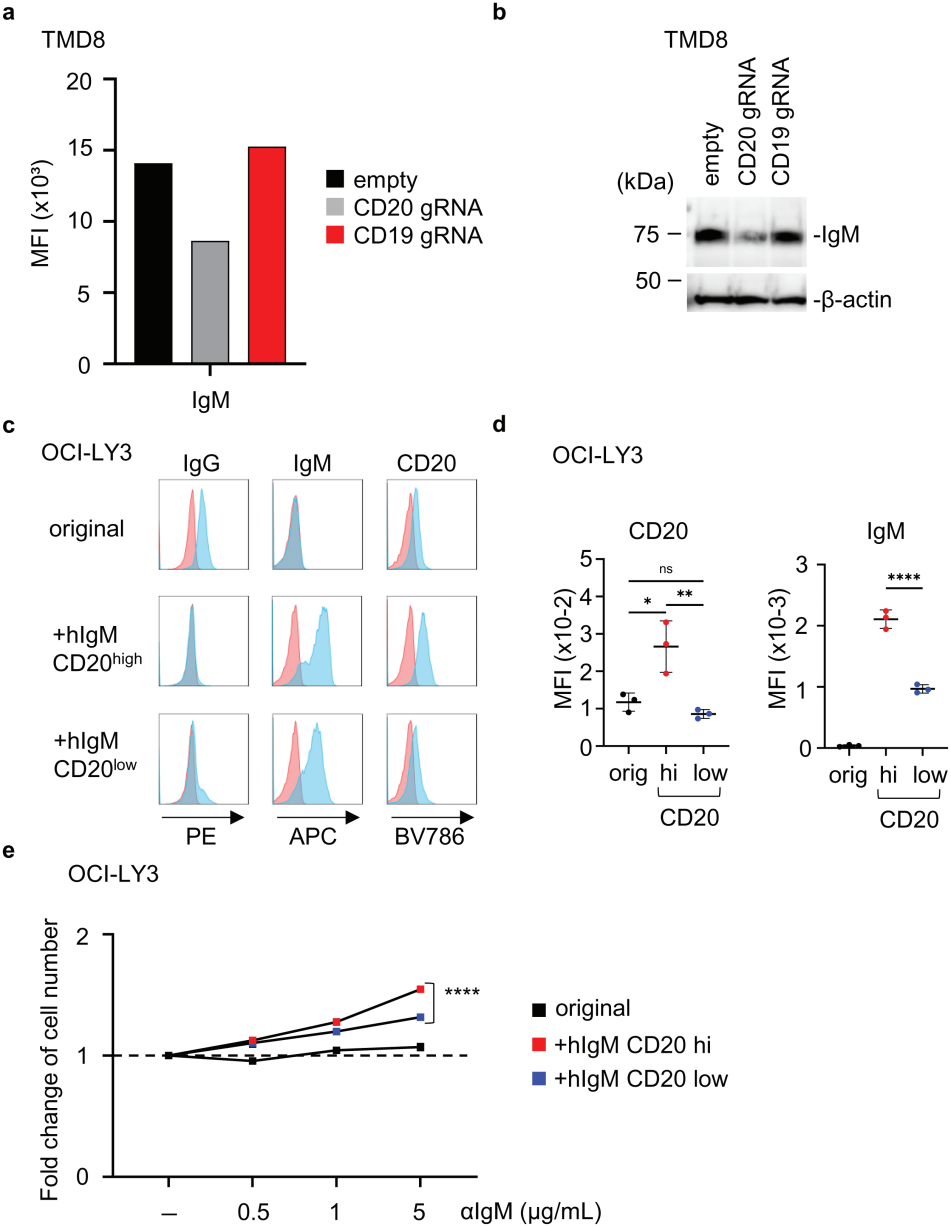
CD20 correlates with cell surface IgM and anti-IgM Ab-dependent proliferation. (a) The mean fluorescence intensity of cell surface IgM on indicated TMD8 lines. (b) Immunoblotting of IgM from indicated TMD8 lines. β-actin is also shown as the loading control. (c) Blue histograms show staining of OCI-Ly3 with the Abs to indicated markers. Red histograms show staining with the isotype control. (d) The mean fluorescence intensity (MFI) values of staining of indicated OCI-LY3 lines with the antibodies to CD20 and IgM. (e) Fold increases in the numbers of indicated OCI-LY3 lines after 2 days of culture with indicated concentrations of anti-IgM Ab. The results are represented by the averaged fold increase ± SEM from triplicates. Dotted lines indicate the fold increase as 1. These experiments were repeated twice, and representative data are shown. *P < 0.05, **P < 0.01, ****P < 0.0001.

We next assessed cell surface IgM expression in parental, CD19-, and CD20-deficient TMD8 lines (Fig. 6a). Similar to observations in Ba/F3 cells, CD20-deficient TMD8 cells exhibited reduced surface IgM expression, whereas CD19-deficient TMD8 cells maintained the levels of surface IgM (Fig. 6a). Consistent with these results, immunoblot analysis revealed a decrease in the amount of IgM protein in CD20-deficient TMD8 cells compared to the parental and CD19-deficient TMD8 lines (Fig. 6b). To confirm and extend these findings, we examined the OCI-LY3 DLBCL line (22), which expressed IgG, but not IgM (Fig. 6c). OCI-LY3 cells were transduced to express human μ heavy chain (OCI-LY3-IgM), and subsequently sorted into CD20^hi^ and CD20^low^ OCI-LY3-IgM populations. Cell surface IgM levels correlated with CD20 expression in OCI-LY3-IgM cells (Fig. 6d). Moreover, IgM ligation promoted proliferation in CD20^hi^ OCI-LY3-IgM more than in CD20^low^ OCI-LY3-IgM (Fig. 6e). These results suggest that CD20 expression positively correlates with cell surface IgM levels and the proliferative response to IgM ligation in the OCI-LY3 line.

### TLR7 and IgM promote survival/proliferation

**Figure 7.**
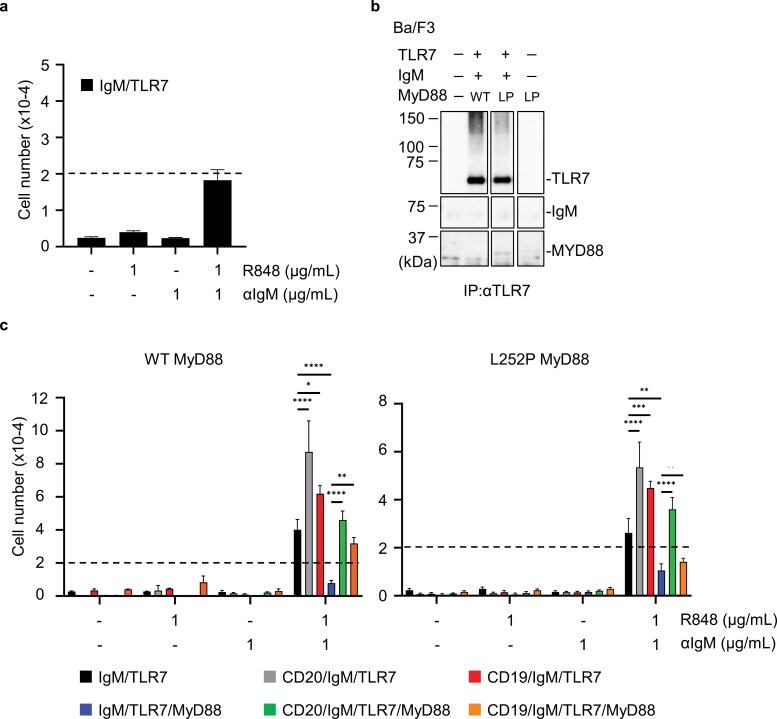
CD19 and CD20 promote TLR7-dependent survival/proliferation. (a) The numbers of IgM/TLR7-expressing Ba/F3 cells that survived the culture with indicated stimulations for 5 days. The dotted line indicates the number of cells at the beginning of the culture. Mean values ± SEM from triplicates are shown. (b) Immunoblot analysis of TLR7, IgM, MyD88 in the immunoprecipitates with anti-TLR7 mAb of indicated Ba/F3 cells. (c) The numbers of indicated Ba/F3 cells that were cultured with indicated stimulations for 5 days. These experiments were repeated twice, and representative data are shown. *P < 0.05, **P < 0.01, ***P < 0.001, ****P < 0.0001.

Finally, we examined the role of TLR7 in IgM-dependent proliferation. TLR7, despite being expressed in B cell lymphoma, does not associate with IgM (2). Nonetheless, TLR7, similar to TLR9, can activate B cells (23). Ba/F3 cells that were transduced to express IgM and TLR7 showed survival and proliferation upon stimulation with anti-IgM antibody and the TLR7 ligand R848 (Fig. 7a). Wild-type MyD88 and L252P MyD88 were additionally expressed and TLR7 was immunoprecipitated to examine TLR7 association with IgM and MyD88. Consistent with the prior report (2), IgM was not co-precipitated with TLR7 (Fig. 7b). In contrast, L252P MyD88, but not wild-type MyD88, was co-precipitated with TLR7 (Fig. 7b), suggesting that TLR7 interacts with L252P MyD88 but not with IgM. We next examined the effect of MyD88 over-expression in TLR7-dependent proliferation of Ba/F3 cells expressing IgM and TLR7. Over-expression of either wild-type or L252P MyD88 downregulated proliferation in response to anti-IgM antibody and R848 (Fig. 7c, compare black and blue). Expression of CD20 or CD19 promoted proliferation in IgM/TLR7-expressing Ba/F3 cells (Supplementary Figure 2, Fig. 7c, compare black, gray, and red) and in IgM/TLR7/wild-type MyD88-expressing Ba/F3 cells (Fig. 7c, compare blue, green, and orange). Furthermore, CD20 expression also enhanced proliferation with anti-IgM and R848 in IgM/TLR7/L252P MyD88-expressing Ba/F3 cells (Fig. 7c, compare blue and green). These results suggest that CD19 and CD20 also promote proliferation driven by TLR7 and IgM probably through mechanisms distinct from those involving TLR9.

## Discussion

We have established the Ba/F3 line capable of proliferating in a manner dependent on IgM, TLR9, and L265P MyD88. Since this Ba/F3 line proliferated either through IL-3 or anti-IgM antibody stimulation, this model system provides a platform to investigate molecular requirements for IgM-dependent proliferation by comparing conditions supported by IL-3 versus anti-IgM antibodies. For example, the selection in the presence of anti-IgM antibody led to increased expression of not only IgM and MyD88 but also CD20. In contrast, such changes were not observed when cells were maintained with IL-3, suggesting that the interaction between IgM and CD20 is essential for IgM-driven proliferation. We believe that the Ba/F3 line established here would contribute to our understanding of molecular mechanisms underlying BCR-dependent proliferation.

CD20 expression showed positive correlation with the amount of cell surface IgM in Ba/F3 cells, TMD8, OCI-LY3. The previous reports showed that CD20^*−*/*−*^ mice exhibit a 20%–30% reduction in IgM expression on immature and mature B cells compared to wild-type mice (9). Similarly, in the B cell lymphoma line Ramos, CD20-deficiency reduces surface IgM as well as other B cell markers, including CD19, CD22, and CD40 (18). As CD20 deficiency in TMD8 specifically diminished surface IgM without altering CD19 levels, CD20 would selectively enhance IgM expression in TMD8. In Ba/F3 cells, we observed CD20-dependent increases in not only cell surface IgM but also in the IgM-TLR9 complex. Increased expression of cell surface IgM is likely to promote the IgM-TLR9 complex formation probably by increasing IgM internalization. Since IgM-ligation disrupts the IgM-CD20 interaction (19), the released IgM might move to endolysosomes to form the IgM-TLR9 complex, instead of being recycled or degraded in lysosomes. In this context, it is worth noting that MS4A4, a homolog of CD20, promotes receptor recycling rather than degradation of the receptor tyrosine kinase KIT in mast cells (24). CD20 may similarly regulate IgM trafficking.

Using the Ba/F3 line, we further demonstrated that CD19 supports IgM-, TLR9-, and L252P MyD88-dependent proliferation by enhancing AKT phosphorylation upon IgM-ligation. Pharmacological inhibition of AKT with MK2206 effectively suppressed Ba/F3 proliferation in the presence of anti-IgM Ab and CpG-B. These findings suggest that CD19 promotes AKT phosphorylation downstream of the BCR in Ba/F3 cells.

In the TMD8 line, concurrent downregulation of CD19 and CD20, rather than CD19 alone, impaired proliferation and AKT phosphorylation in response to IgM ligation. Notably, CD19/CD20-deficient TMD8 exhibited increased resistance to the AKT inhibitor MK2206 compared to parental TMD8 cells, suggesting that both CD19 and CD20 promote AKT-dependent proliferation. Although a direct CD20-mediated enhancement of IgM-induced AKT-phosphorylation was not observed in Ba/F3 cells, the CD20-dependent increase in cell surface IgM and the IgM-TLR9 complex likely facilitates AKT-phosphorylation after IgM-ligation. These findings suggest that CD19 and CD20 support AKT-dependent proliferation in TMD8 cells through distinct mechanisms (Supplementary Figure 3).

B cells express TLR7 and respond to the TLR7 ligands. TLR7 activation has been implicated in the expansion of RNA-specific B cells in lupus-prone mice as well as monogenic lupus in humans (25–27). Unlike TLR9, TLR7 did not associate with IgM, consistent with the previous report (2). Although L252P MyD88 interacted with TLR7, it is unlikely that the IgM-TLR7-MyD88 complex forms. Since anti-IgM antibody and the TLR7 ligand R848 cooperatively enhance Ba/F3 cell proliferation, the downstream signals triggered by IgM and TLR7 might interact with each other.

## Supplementary data

Supplementary data are available at *International Immunology* Online.

dxaf004_suppl_Supplementary_Figure_S1-S3
