# Supplementary material for: CD20 and CD19 promote proliferation driven by the IgM-TLR9-L265P MyD88 complex
Source: Int Immunol. 2025 Jan 27;37(6):325–37. doi: 10.1093/intimm/dxaf004 (PMC12096165; doi:10.1093/intimm/dxaf004)
Supplement: dxaf004_suppl_Supplementary_Figure_S1-S3 [file dxaf004_suppl_supplementary_figure_s1-s3.docx]

**Fig. S1. FACS analyses of Ba/F3 lines used for the TLR9 study**

1. Blue and histograms show staining with the antibodies to indicated

molecules and isotype-matched control Abs, respectively. (b) Histograms show GFP expression in indicated Ba/F3 cells that expressed the NF-κB-GFP reporter plasmid. These Ba/F3 cells were left unstimulated.

**
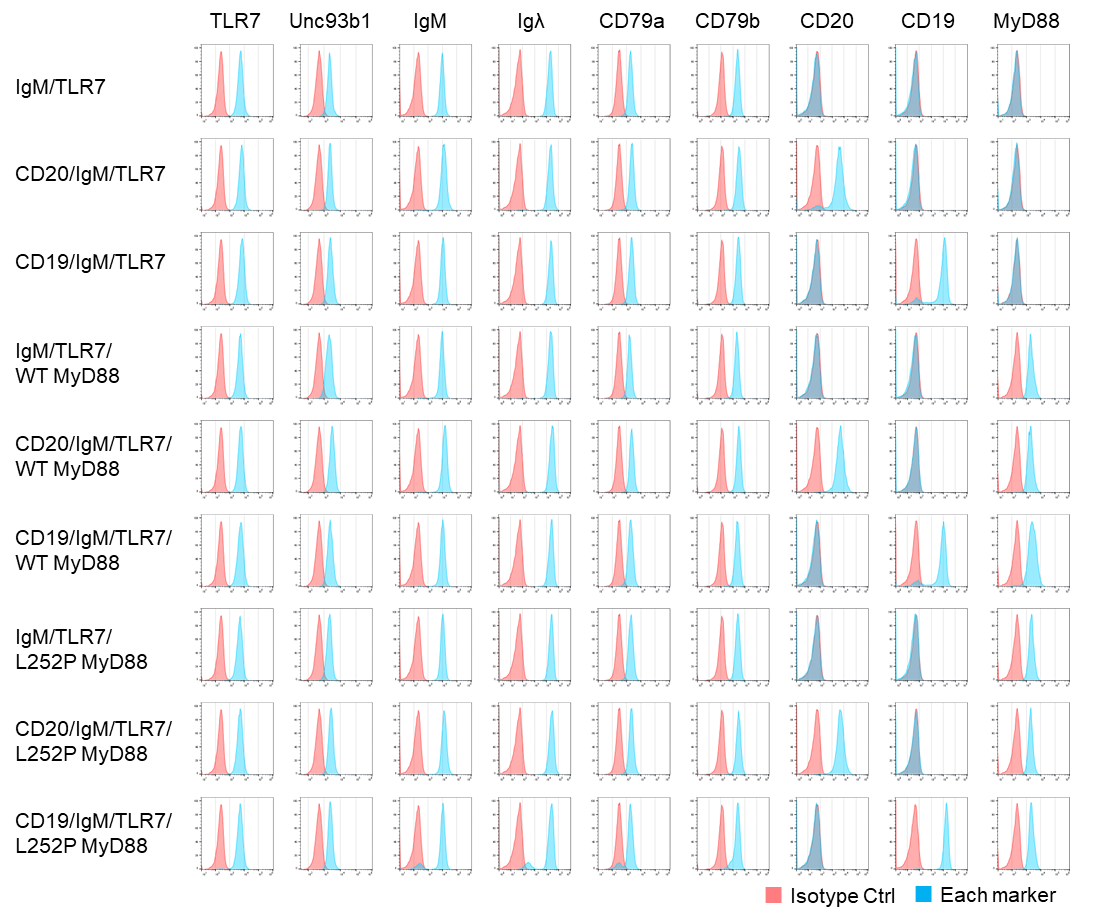
**

**Fig. S2. FACS analyses of Ba/F3 lines used for the TLR7 study**

Blue and histograms show staining with the antibodies to indicated molecules and isotype-matched control Abs, respectively.

**
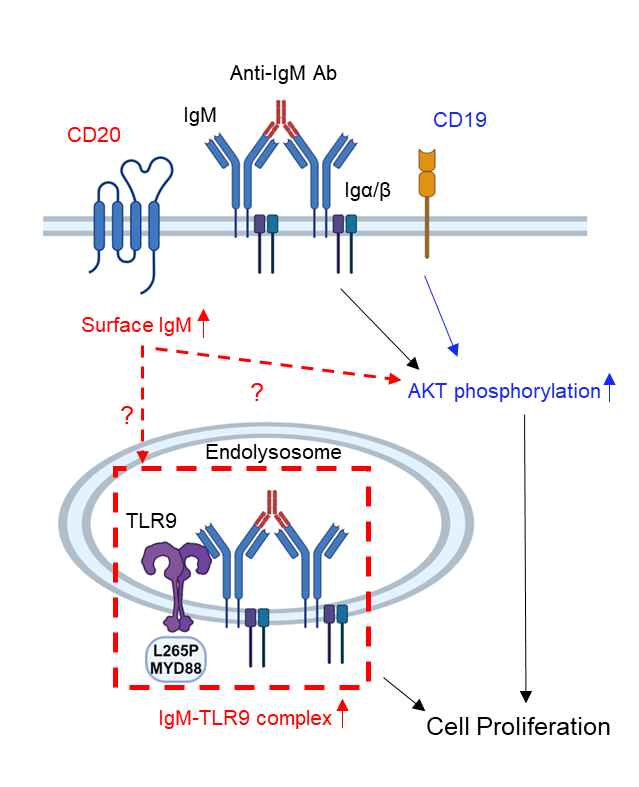
**

**Fig. S3. CD19 and CD20 differentially contribute to the proliferation driven by the My-T-BCR complex**

CD20 expression increases the amount of cell surface IgM and thereby the IgM-TLR9 complex in the endosomal compartment, leading to enhanced proliferation. CD19 augments IgM-dependent AKT-phosphorylation in Ba/F3 cells. In TMD8, both CD19 and CD20 enhance IgM-dependent AKT-phosphorylation.
